# Constitutive expression of NF-κB inducing kinase in regulatory T cells impairs suppressive function and promotes instability and pro-inflammatory cytokine production

**DOI:** 10.1038/s41598-017-14965-x

**Published:** 2017-11-07

**Authors:** Fanny Polesso, Minhazur Sarker, Arian Anderson, David C. Parker, Susan E. Murray

**Affiliations:** 1 Department of Molecular Microbiology and Immunology, Oregon Health & Science University, Portland, OR 97239 USA; 2 Department of Biology, University of Portland, Portland, OR USA

## Abstract

CD4^+^Foxp3^+^ regulatory T cells (Tregs) are indispensable negative regulators of immune responses. To understand Treg biology in health and disease, it is critical to elucidate factors that affect Treg homeostasis and suppressive function. Tregs express several costimulatory TNF receptor family members that activate non-canonical NF-κB via accumulation of NF-κB inducing kinase (NIK). We previously showed that constitutive NIK expression in all T cells causes fatal multi-organ autoimmunity associated with hyperactive conventional T cell responses and poor Treg-mediated suppression. Here, we show that constitutive NIK expression that is restricted to Tregs via a Cre-inducible transgene causes an autoimmune syndrome. We found that constitutive NIK expression decreased expression of numerous Treg signature genes and microRNAs involved in Treg homeostasis and suppressive phenotype. NIK transgenic Tregs competed poorly with WT Tregs *in vivo* and produced pro-inflammatory cytokines upon stimulation. Lineage tracing experiments revealed accumulation of ex-Foxp3^+^ T cells in mice expressing NIK constitutively in Tregs, and these former Tregs produced copious IFNγ and IL-2. Our data indicate that under inflammatory conditions in which NIK is activated, Tregs may lose suppressive function and may actively contribute to inflammation.

## Introduction

Foxp3^+^ regulatory CD4 T cells (Tregs) are indispensable immune regulators. Genetic lesions in Foxp3 or experimental depletion of Tregs causes lethal multi-organ autoimmunity in mice and humans^1^. Like other T cell subsets, Tregs are activated through TCR engagement by peptide-MHC complexes. TCR activation in Tregs, however, leads to immunosuppressive rather than pro-inflammatory functions. Tregs express a TCR repertoire skewed towards self and commensal bacterial antigens^2–6^; thus, their phenotypic stability is paramount lest they become pathogenic themselves. Although controversy exists as to the degree of Treg stability under homeostatic and inflammatory conditions^7–9^, it is clear that under certain circumstances they can lose suppressive function, at least temporarily^10–16^. Relieving Treg-mediated suppression permits effective immune responses to clear pathogens or cancer cells^11,17,18^, but impaired Treg homeostasis and function is associated with inflammation and autoimmunity^7,19,20^.

NIK (MAP3K14) is an essential kinase that links several co-stimulatory TNF receptor family members (TNFRs) to non-canonical NF-κB activation. These receptors include TNFR2, TNFRSF4 (CD134, OX40), TNFRSF18 (GITR), and TNFRSF9 (CD137, 4-1BB), which all have been implicated in decreasing Treg function or phenotypic stability^21–29^. However, conflicting reports have shown instances in which these receptors can increase Treg numbers and/or suppressive function^27,30–34^. It has been difficult to tease out mechanisms that may account for these discrepancies, in part because TNFR ligation recruits TRAFs that can activate diverse kinases including ERK1/2, PI3K/AKT, TAB/TAK, IKK complex, and NIK^35^. There is a need to parse the effects of individual intracellular signaling pathways downstream of TNFRs to identify common targets for immunotherapy that aims to turn Tregs off or on.

We previously found that constitutive expression of NIK in all T cells impairs Treg function^36^. In addition, NIK was recently identified as a multiple sclerosis susceptibility gene in a genome-wide association study^37^. Moreover, aberrations in the non-canonical NF-κB pathway downstream of NIK can lead to autoimmunity in mice^36,38–42^. Despite this growing evidence that aberrant signaling downstream of NIK in effector T cells can contribute to autoimmune pathogenesis, the effect of NIK on Treg function is unknown.

To investigate the role of NIK in Treg function, we used mice carrying an inducible, constitutively expressed NIK transgene. When we restricted NIK transgene expression to Tregs, mice developed an autoimmune phenotype characterized by poorly suppressive Tregs. Mechanistically, NIK overexpression altered Treg signature gene expression, impaired Treg phenotypic stability, and de-repressed pro-inflammatory cytokine production by Tregs.

## Results

### NIK intrinsically impairs Treg function *in vitro* and *in vivo*

**Figure 1:**
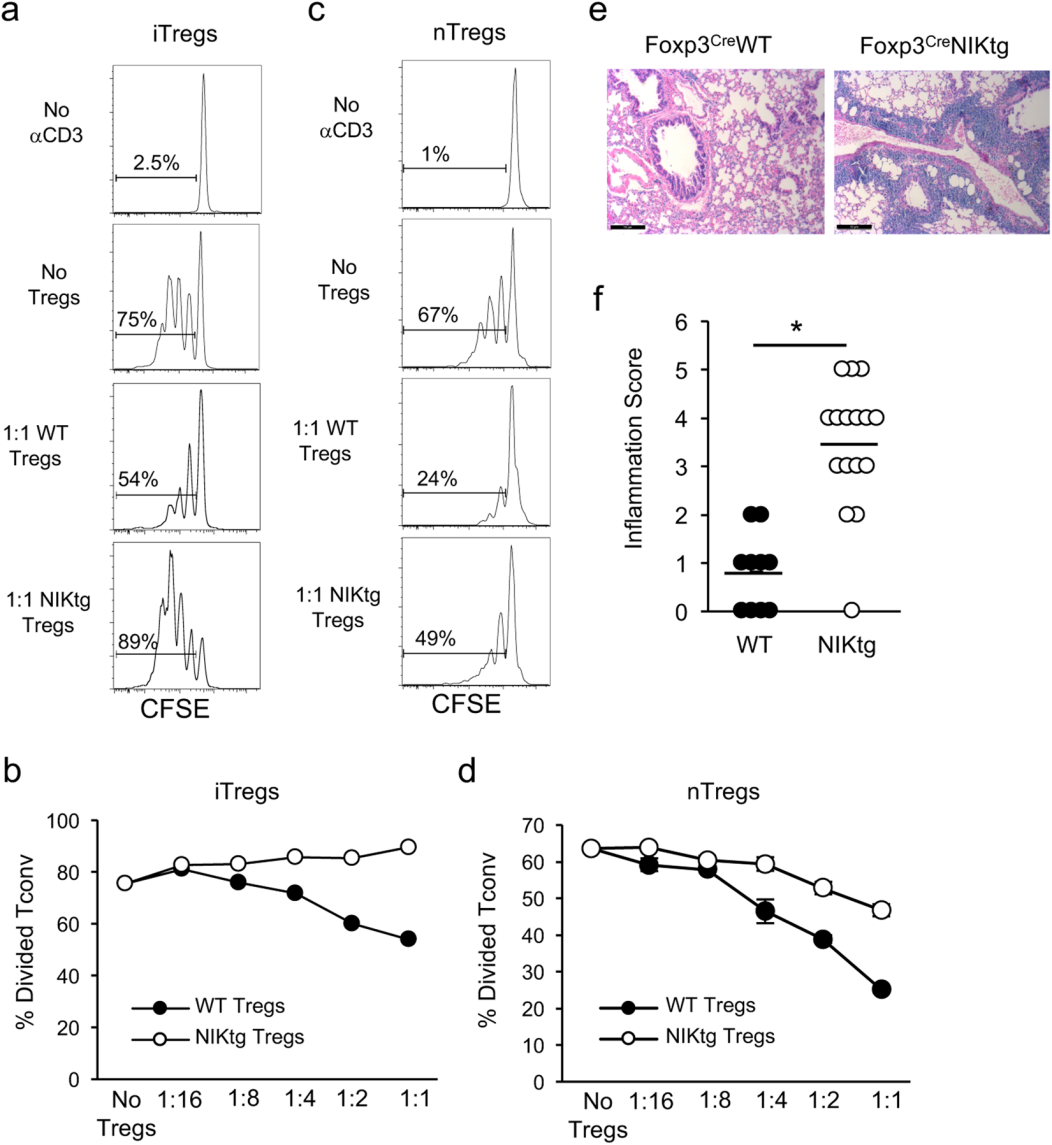
NIK upregulation impairs Treg suppressive function *in vitro* and *in vivo*. (**a**,**b**) iTregs: Purified NIKtg/Foxp3^RFP^ or WT/Foxp3^RFP^ CD4 Tconv were treated with TAT-Cre fusion protein to induce expression of the NIK transgene and were cultured under iTreg inducing conditions. Four days later, Foxp3^RFP+^ iTreg cells were sorted and assessed for suppressive function against CFSE-labeled WT CD4^+^ Tconv. (**a**) Histograms depict CFSE intensity of gated WT CD4^+^ Tconv; numbers indicate percent of WT CD4 Tconv that have divided at least once. (**b**) X-axis indicates Treg: Tconv ratios; y-axis indicates percent of WT CD4^+^ Tconv that have divided at least once as determined by gating as in (**a**). (**c**,**d**) nTregs: CD4^+^RFP^+^ WT and NIKtg nTregs were sorted directly from spleens of mixed BM chimera recipients that had been reconstituted with equal numbers of NIKtg/CD4^Cre^/Foxp3^RFP^ and WT/Thy1.1/Foxp3^RFP^ bone marrow cells. The sorted NIKtg and WT nTregs were assessed for suppressive function against WT CD4^+^ Tconv as in (**a**,**b**). (**a–d)** Data are from one representative experiment of 2; replicate data are shown in Supplementary Fig. S1. (**e**,**f**) Lungs from NIKtg/Foxp3^Cre^ and WT/Foxp3^Cre^ littermates were assessed for immunopathology by H&E staining. Data in (**f**) are pooled from 3 independent cohorts of mice; each data point represents the score of an individual mouse *p < 0.05.

NIK transgenic (NIKtg) mice harbor a single copy NIK^fl-STOP-fl-GFP^ transgene knocked into the ROSA-26 locus. Cre expression excises the floxed STOP, allowing co-expression of NIK and GFP, via an IRES. We previously showed that T cell restricted constitutive NIK expression in CD4^Cre^/NIKtg mice activates non-canonical NF-κB in T cells and causes early onset lethal multi-organ autoimmunity^36^. In that study, we sorted conventional T cells (Tconv) and Tregs based on CD4 and CD25 expression and found that constitutive NIK expression exerts cell-intrinsic effects on both T cell subsets that, in combination, impair Treg suppressive function. In order to test the suppressive function of more highly purified *in vitro* generated Tregs (iTregs), we sorted CD4^+^ Tconv from NIKtg/Foxp3^RFP^ and WT/Foxp3^RFP^ littermate control mice and cultured them in Treg-inducing conditions. During culture, we induced NIK transgene expression via protein transduction with TAT-Cre, which recombines the NIK^fl-STOP-fl-GFP^ locus at ~60% frequency. After 3 days, we sorted NIKtg and WT Tregs (CD4^+^GFP^+^RFP^+^ and CD4^+^GFP^−^RFP^+^, respectively) and assessed their ability to suppress WT CD4 Tconv cell proliferation. Consistent with our prior report, we found that NIK expression intrinsically impaired the ability of iTregs to suppress Tconv cell proliferation (Fig. 1a,b and Supplementary Fig. S1). We also assessed whether NIKtg natural Tregs (nTregs) had impaired suppressive function. Mixed bone marrow (BM) chimera recipients were reconstituted with equal numbers of BM precursors from CD4^Cre^/NIKtg/Foxp3^RFP^ and Thy1.1/WT/Foxp3^RFP^ mice. Unlike CD4^Cre^/NIKtg mice, in which nearly all T cells express the NIK transgene, only half of the T cells in mixed BM chimeras express the NIK transgene. These mice remain healthy and afford us the opportunity to compare NIKtg and WT Tregs isolated from the same environment^36^. This ensured that we were measuring cell-intrinsic differences rather than differences secondary to an inflammatory environment. From these BM chimeras, we sorted NIKtg and WT Tregs directly *ex vivo* to >98% purity (Supplementary Fig. S2) and assessed their ability to suppress WT CD4 Tconv cell proliferation. Although the NIKtg nTregs exerted modest suppression, it was much less than that of WT Tregs (Fig. 1c,d and Supplementary Fig. S1).

To test whether NIKtg Tregs are poor suppressors of inflammation *in vivo*, we generated mice in which the NIK transgene is induced by Foxp3^Cre^. These mice did not succumb to the rapid, pre-weaning multi-organ autoimmunity seen in NIKtg/CD4^Cre^ mice^36^, but a small proportion died between 6 and 8 months. Necropsy revealed severe lung inflammation, but no other organ pathology (Fig. 1e). Upon euthanasia, most of the other NIKtg mice had also developed moderate to severe lung inflammation (Fig. 1f). Thus, NIK overexpression in Tregs alone is sufficient to cause autoimmunity.

### Microarray and microRNA array gene expression patterns in NIKtg vs. WT Tregs

**Figure 2:**
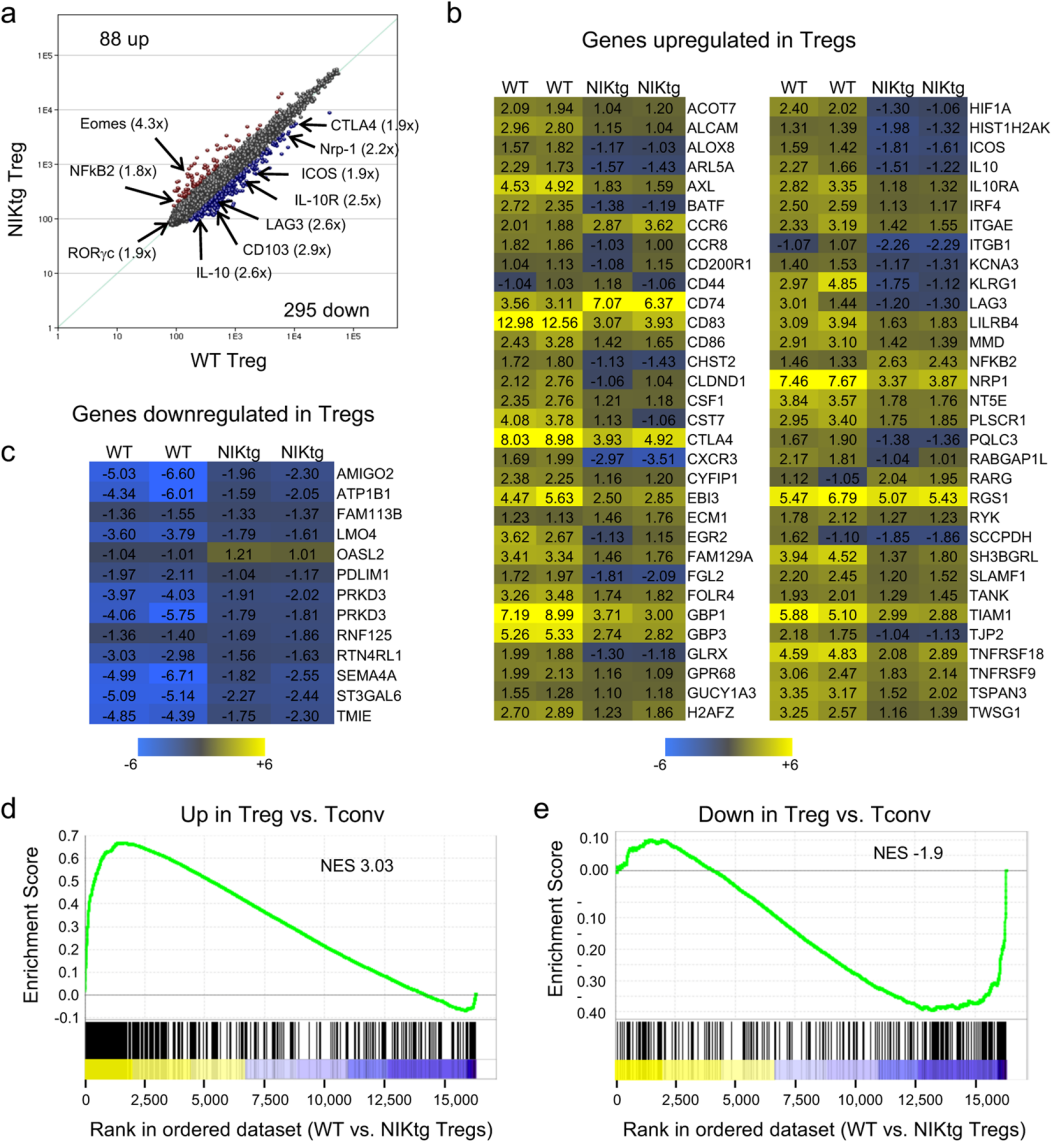
Gene expression patterns in NIKtg Tregs. WT and NIKtg Tregs (CD4^+^CD25^hi^) were sorted from spleens of healthy mixed BM chimeric mice in which WT BM chimeric mice were reconstituted with equal numbers of CD4^Cre^/NIKtg and congenically marked WT bone marrow. Total RNA was isolated for microarray and miRNA analysis (Fig. 3). Data show averages of 2 biological replicates for each population except (**b**,**c)**, which show both replicates. (**a**) Correlation of gene expression between WT and NIKtg Tregs. (**b**,**c**) Genes classically increased (**b**) or decreased (**c**) in Tregs (Immgen) that are differentially regulated between NIK-tg and WT Tregs. Numbers indicate fold change versus WT Tconv. (**d**,**e**) GSEA analysis using Immgen generated “upregulated in Tregs vs. Tconv” and “downregulated in Tregs vs. Tconv” gene sets. Genes are ranked along the x-axis by their fold change in WT Tregs vs. NIKtg Tregs with yellow indicating genes higher in WT Tregs and blue indicating genes lower in WT Tregs (higher in NIKtg Tregs). Enrichment scores are indicated on the y-axis; negative enrichment score (**e**) indicates that the gene set is enriched in those genes that are higher in NIKtg Tregs than WT Tregs. NES, normalized enrichment score. For both plots, the indicated gene set is significantly enriched at nominal p-value < 0.01 and nominal FDR < 0.25.

We examined how chronic NIK expression affects global gene expression patterns using microarray and miRNA array analyses on RNA isolated from NIKtg and WT Tregs. Again, we sorted these cells from mixed bone marrow chimeras reconstituted with equal proportions of CD4^Cre^/NIKtg BM and congenically marked WT BM to ensure that we were measuring cell-intrinsic differences. We also sorted CD4^+^ Tconv from these mice as a reference point. Using a 1.8-fold difference cutoff, we found 295 genes downregulated and 88 genes upregulated in NIKtg Tregs compared to WT Tregs (Fig. 2a). Several of the downregulated genes encode Treg effector molecules, such as CTLA-4, IL-10, LAG3, CD44, ICOS, and neuropilin-1. In addition to genes encoding Treg effector molecules, downregulated genes included cytokine and homing receptors (*Il10r, Cd103, Cxcr3*) and transcription factors (*Hif1a, Irf4*) that have been implicated in Treg function and fitness. However, consistent with our ability to sort these cells based on Foxp3^RFP^ expression, *Foxp3* itself was not different between NIKtg and WT Tregs (Supplementary Fig. S3). Moreover, NIKtg Foxp3^+^ T cells in these chimeras are clearly bona fide Tregs as assessed by their expression of the Treg markers, CD25, CTLA-4, CD39, and Helios (Supplementary Fig. S3). Although NIKtg Treg expressed somewhat lower levels of CD25 and CTLA-4 than WT Tregs, these markers were still much higher on NIKtg Tregs than on WT or NIKtg Tconv (Supplementary Fig. S3).

We compared our list of genes that differed between NIKtg and WT Tregs with the list of genes that differ between CD4^+^Foxp3^GFP+^ (Treg) and CD4^+^Foxp3^GFP−^ (Tconv) populations provided by Mathis and Benoist in the Immgen database^43,44^. Overall, NIKtg Tregs have a gene expression pattern consistent with identity as Tregs—of 832 total Treg signature gene changes determined by Immgen analysis, only 77 (9%) differed between NIKtg and WT Tregs (Fig. 2b,c). However, those Treg signature genes that did differ between NIKtg and WT Tregs revealed an interesting pattern. Genes typically upregulated in Tregs vs. Tconv tended to show lower expression in NIKtg vs. WT Tregs, as depicted by less intense yellow color or blue color on the heat map (Fig. 2b). Furthermore, of genes typically downregulated in Tregs, those differentially expressed between NIKtg and WT Tregs were all higher in NIKtg Tregs vs. WT Tregs, as depicted by less intense blue color or yellow color on the heat map (Fig. 2c).

To statistically analyze the expression of Treg signature genes in our microarray, we used the Treg signature gene sets generated by Immgen described above to perform gene-set enrichment analysis (GSEA)^45,46^. Genes that are upregulated in Tregs vs. Tconv were significantly enriched among genes we found to be regulated when comparing WT vs. NIKtg Tregs (Fig. 2d). Conversely, genes that are downregulated in Tregs vs. Tconv showed a negative enrichment score in WT vs. NIKtg Tregs, which is equivalent to positive enrichment in a NIKtg vs. WT comparison (Fig. 2e). These data show that constitutive NIK expression in Tregs tends to decrease genes that are usually upregulated in Tregs and increases genes that are downregulated in Tregs. Essentially, despite normal Foxp3 expression and an overall gene pattern consistent with a Treg phenotype, these results show that NIKtg Tregs are less “Treg-like” than WT Tregs, which could indicate that constitutive NIK expression destabilizes the Treg phenotype.

**Figure 3:**
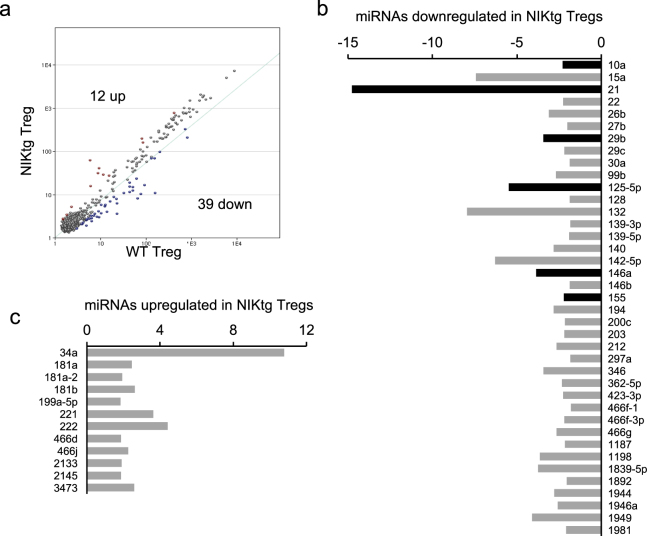
miRNA expression patterns in NIKtg Tregs. The same samples of RNA were used for miRNA analysis as for microarray analysis shown in Fig. 2. (**a**) Correlation of miRNA expression between WT and NIKtg Tregs. (**b**,**c**) miRNAs that are decreased (**b**) or increased (**c**) in NIKtg Tregs relative to WT Tregs. X-axes indicate fold-change in miRNA expression; y-axes indicate individual miRNAs. Black bars in (**b**) are shown in Table 1.

We also compared miRNA gene expression between NIKtg and WT Tregs. miRNA-mediated regulation is critical for Treg function and homeostatic potential as evidenced by severe autoimmunity upon Treg-specific deletion of the miRNA processing components Drosha and Dicer^47,48^. We found 51 miRNAs that differed between NIKtg and WT Tregs—39 were decreased in NIKtg, and 12 were increased (Fig. 3a–c). Six of these differentially expressed miRNAs are known to promote Treg function or homeostatic maintenance, and all 6 were downregulated in NIKtg Treg vs. WT Tregs (Fig. 3c, Table 1). Together, these 6 miRNAs have been shown to repress IFNγ receptor signaling and IFNγ production, maintain Foxp3 expression via repression of genes that inhibit Foxp3 expression, and increase Treg survival signals mediated by IL-2R signaling via repression of negative regulators of this pathway (Table 1). Thus, the miRNA landscape suggests that constitutive NIK expression may decrease Treg survival and impair Treg phenotypic stability.

**Table 1:** Treg intrinsic roles of microRNAs that are differentially expressed between NIKtg and WT Tregs.

miRNA	Fold change^a^	Targets described in T cells	Predicted effect of decreased miR in Tregs	Citations
miR-21	14.8 down	Dnmt1, SATB1	Loss of Foxp3, acquisition of T_eff_ phenotype	68,84,85
miR-125a	5.5 down	STAT3, IFNγ, IL-13	Decreased suppressive function, acquisition of T_eff_ phenotype	71
miR-146a	3.9 down	STAT1 signaling	Production of IFNγ	67
miR-29b	3.4 down	IFNγ	Production of IFNγ	72–74
miR-10a	2.3 down	Bcl-6	Decrease in Foxp3	69,70
miR-155	2.2 down	SOCS1, SATB1	Decreased CD25-mediated survival, acquisition of T_eff_ phenotype	85,86

^a^fold change in expression levels, NIKtg Tregs vs. WT Tregs.

### NIK intrinsically alters Treg proportions and phenotype in mixed bone marrow chimeras

**Figure 4:**
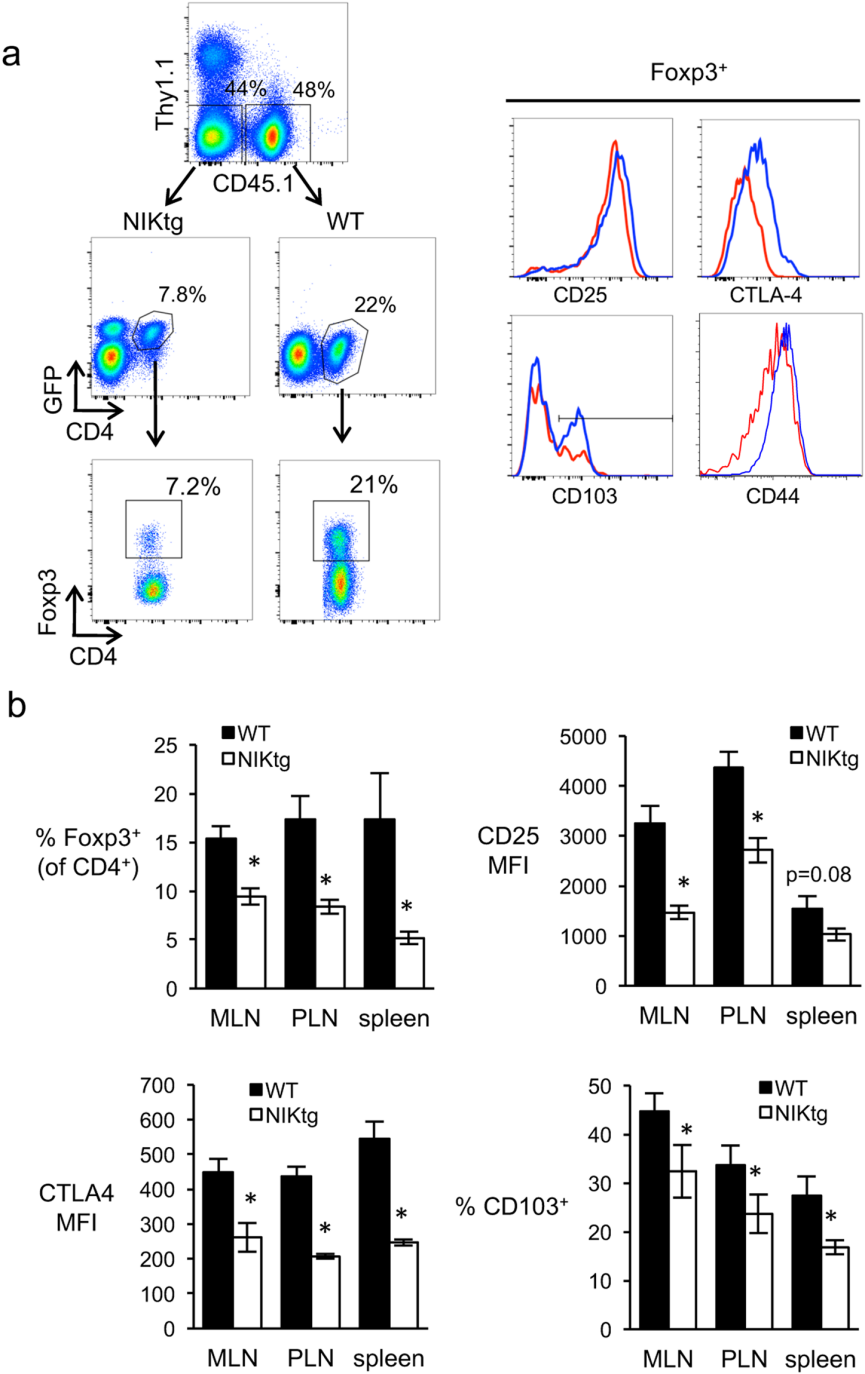
NIKtg Treg proportions and phenotype in mixed BM chimeras. Mixed BM chimeric mice were generated as described in Fig. 2 except that mixed BM recipients were Thy1.1 and WT BM was CD45.1. (**a**) Gating strategy and representative histograms of surface molecule expression in splenocytes. Upper left dot plot is gated on live lymphocytes. Blue lines, WT; red lines, NIKtg. (**b**) Quantitation of Foxp3^+^ proportion within the indicated genotype of CD4 T cells (upper left graph) and expression of CD25, CTLA-4, and CD103 on Foxp3^+^ gated cells from the indicated organs. MFI, mean fluorescence intensity; MLN, mesenteric lymph nodes; PLN, peripheral lymph nodes. Data are from one representative experiment of 3. Bar graphs depict mean +/− SD (n = 5 mice). *p < 0.05.

In the course of sorting Treg and Tconv cell populations for the Treg suppression assays and microarray experiments, we noted that the ratio of Tregs: Tconv was lower in NIKtg than WT T cell populations within mixed BM chimeras. To confirm this difference, we quantified relative proportions of Tregs in mixed BM chimeras and found a 2–3 fold decrease in the proportion of Tregs within the NIKtg CD4 compartment compared to the WT CD4 compartment (Fig. 4a,b). The phenotype of the NIKtg Tregs also differed from that of WT Tregs in mixed BM chimeras. In validation of our microarray results, the protein expression level of Foxp3 itself was not different between NIKtg and WT Tregs; however, CTLA-4, CD44, and CD103 protein levels were decreased in NIKtg Tregs, as was CD25 expression (Fig. 4a,b). These changes were consistent among peripheral lymph nodes, mesenteric lymph nodes, and spleen (Fig. 4b). These data support the hypothesis that constitutive NIK expression impairs Treg homeostatic potential and alters their phenotype.

### NIK antagonizes IL-2 mediated iTreg expansion and disinhibits proinflammatory cytokine production by Tregs *in vitro* and *in vivo*

The decreased proportion and altered gene expression of NIKtg Tregs in mixed BM chimeras suggested to us that constitutive NIK expression might intrinsically render Tregs phenotypically unstable, allowing them to convert to an effector phenotype under inflammatory conditions. To test this, we first sorted CD4^+^Foxp3^RFP+^ WT and NIKtg Tregs from mixed BM chimeras and tested their ability to retain Foxp3 expression under various *in vitro* conditions: TCR stimulation alone or with addition of IL-2, IL-6, APCs, or WT Tconv. Over the course of 3–6 days, there was no difference in maintenance of Foxp3 expression between NIKtg and WT nTregs under any of these conditions (Supplementary Fig. S4).

**Figure 5:**
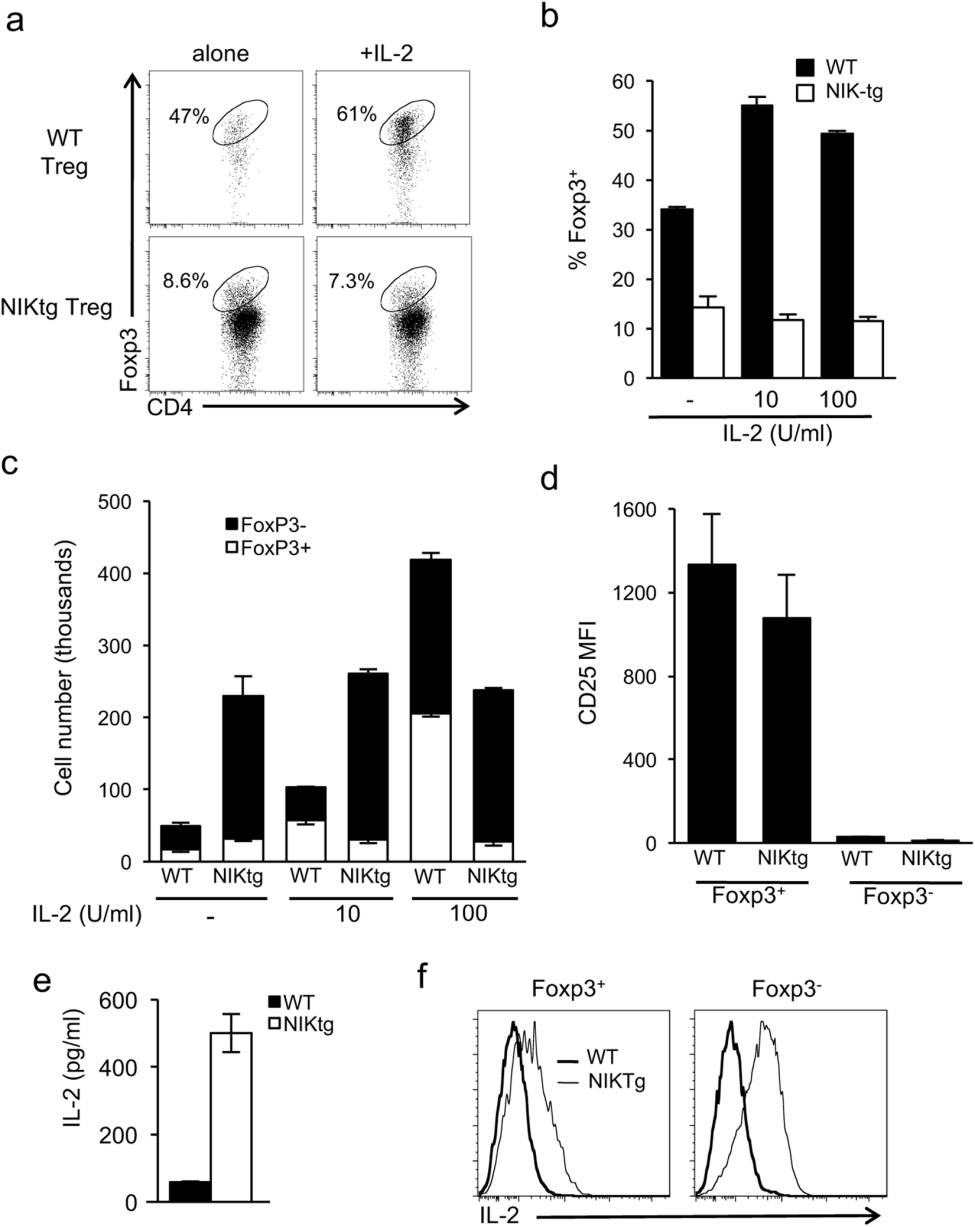
Altered IL-2 responsiveness and IL-2 production by NIKtg Tregs. CD4^+^ Tconv from NIKtg/Foxp3^RFP^ and WT/Foxp3^RFP^ mice were treated with TAT-Cre and cultured under Treg-inducing conditions as in Fig. 1. After 3 days, CD4^+^Foxp3^RFP+^ cells were sorted and recultured either alone or with additional IL-2. After 3 days of secondary culture, cells were assessed for maintenance of Foxp3 expression. (**a**) Representative flow cytometry plots gated on live lymphocytes. (**b**) Percent Foxp3^+^ T cells in secondary cultures supplemented with the indicated concentrations of recombinant IL-2. (**c**) Total number of Foxp3^+^ and Foxp3^−^ cells in secondary cultures supplemented with the indicated concentrations of recombinant IL-2. (**d–f**) Cells not treated with IL-2 in secondary culture were assessed for CD25 expression (**d**) and IL-2 production as measured by ELISA on the culture supernatant (**e**) and by intracellular cytokine stain upon restimulation with PMA + ionomycin (**f**). Data are from one representative experiment of 3 (**a–d**) or one representative experiment of 2 (**e**,**f**); replicate data are shown in Supplementary Fig. S5. Bar graphs depict mean +/− SD of triplicate culture wells.

We did, however, find a difference in Foxp3 maintenance between NIKtg and WT iTregs generated *in vitro*. We purified Tconv from NIKtg-Foxp3^RFP^ mice and cultured them in Treg-inducing conditions following TAT-Cre treatment to mediate NIK transgene expression as in Fig. 1a,b. After 3 days, we sorted CD4^+^Foxp3^RFP+^ NIKtg and WT iTregs and recultured them for an additional 3 days. After this secondary culture period, a significantly smaller proportion of NIKtg T cells remained Foxp3^+^ compared with WT T cells (Fig. 5a,b). This skewed ratio appeared to be an effect of increased numbers of Foxp3^−^ cells in the culture, rather than decreased numbers of Foxp3^+^ cells (Fig. 5c, first set of bars).

IL-2 is an essential survival and growth factor for Tregs, and it acts as an important regulatory circuit by increasing the ratios of Tregs to effectors T cells that typically produce IL-2^49^. We asked whether exogenous IL-2 could restore normal regulation to NIKtg iTreg cultures. As expected, IL-2 increased proportions and numbers of WT iTregs, but it had no effect on proportions or numbers of NIKtg iTregs (Fig. 5a–c). We thought decreased CD25 expression on NIKtg Tregs might explain the lack of effect of exogenous IL-2, but unlike NIKtg nTregs directly *ex vivo*, NIKtg iTregs generated *in vitro* expressed normal levels of CD25 (Fig. 5d). The high numbers of Foxp3^−^ Tconv in NIKtg secondary cultures in the absence of exogenous IL-2 (Fig. 5c, left bars) suggested a cellular source of IL-2. Supernatant from cultures without exogenous IL-2 showed high levels of IL-2 in NIKtg but not WT cultures (Fig. 5e and Supplementary Fig. S5). In addition, intracellular cytokine staining showed that in secondary NIKtg T cell cultures both Foxp3^+^ and Foxp3^−^ cells produced IL-2 (Fig. 5f and Supplementary Fig. S5). Thus, despite producing their own IL-2, IL-2 does not provide a survival advantage to NIKtg Tregs, which may upset normal negative feedback mechanisms.

**Figure 6:**
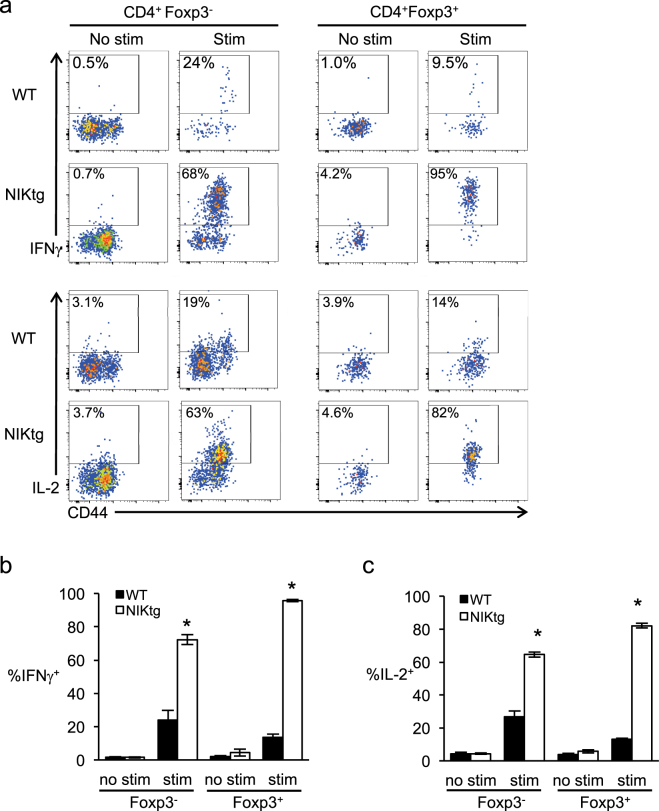
NIKtg Tregs make pro-inflammatory cytokines *in vivo*. Mixed BM chimera receipients were reconstituted with a 50:50 mix of CD4^Cre^xNIKtg BM and congenically marked WT BM in order to directly compare NIKtg with WT T cells in both the Treg and Tconv compartments in the same host. Spleens were harvested from these mixed BM chimeras between 8 and 16 weeks post reconstitution. Treg and Tconv from the spleens were assessed for intracellular IFNγ and IL-2 production upon 5 hours of PMA + ionomycin stimulation. (**a**) Representative flow cytometry plots, gated as indicated after gating on live lymphocytes. (**b**,**c**) Proportion of CD4^+^Foxp3^+^ and CD4^−^Foxp3^−^ T cells within CD4^Cre^xNIKtg and WT T cell populations that produced IFNγ (**b**) and IL-2 (**c**). Data are from one representative experiment of 2; replicate data are shown in Supplementary Fig. S6. Bar graphs depict average +/− SD (n = 5 mice). *p < 0.05.

A hallmark of Tregs is Foxp3-mediated suppression of pro-inflammatory gene transcription, including IL-2^50–53^, so it was surprising that the Foxp3^+^ population of cultured NIKtg iTregs made IL-2 upon TCR stimulation. To determine if constitutive NIK expression *in vivo* endows nTregs with the capacity to produce pro-inflammatory mediators, we assessed IL-2 and IFNγ production directly *ex vivo* in T cells from mixed BM chimeras. Approximately 25% of WT Tconv (CD4^+^Foxp3^−^) produced IFNγ and/or IL-2 upon stimulation with PMA + ionomycin, and these resided in the CD44^hi^ memory compartment (Fig. 6). Fewer than 15% of WT Tregs produced these cytokines upon stimulation. In contrast, nearly three quarters of NIKtg Tconv produced IFNγ and/or IL-2, and over 90% of NIKtg Tregs did so (Fig. 6 and Supplementary Fig. S6). Thus, NIK expression intrinsically allows Tregs to produce pro-inflammatory cytokines despite Foxp3 expression.

### Lineage tracing reveals expansion of inflammatory ex-Foxp3^+^ cells when NIK overexpression is Treg restricted

**Figure 7:**
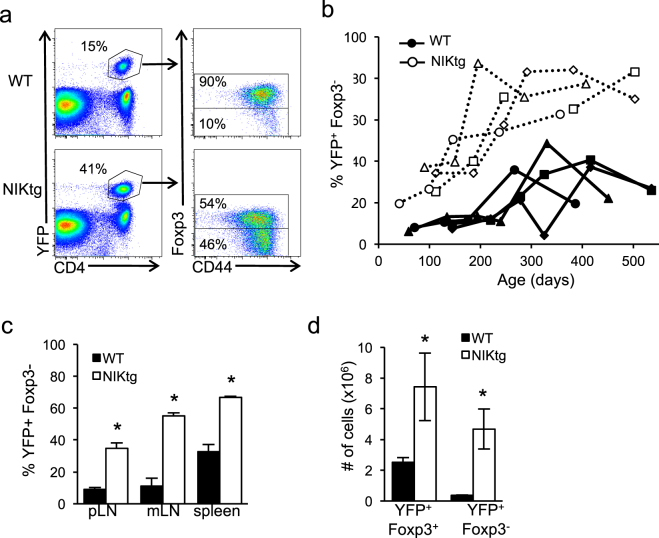
Constitutive NIK expression expands ex-Foxp3^+^ T cells *in vivo*. CD4 T cells from NIKtg/Foxp3^Cre^/R26^YFP^ and WT/Foxp3^Cre^/R26^YFP^ littermates were assessed for percent ex-Foxp3^+^ T cells (defined as percent of total CD4^+^YFP^+^ cells that are Foxp3^−^). (**a**) Representative FACS plots from blood showing the gating scheme. (**b**) Percent ex-Foxp3^+^ T cells in blood over time. Each symbol and line represents an individual mouse. (**c**,**d**) Percent and number of ex-Foxp3^+^ T cells in indicated organs at euthanasia. mLN, mesenteric lymph nodes; pLN, peripheral lymph nodes. All data are from one representative experiment of 3. Bar graphs depict means +/− SD (n = 4 mice per group). *p < 0.05.

In severe inflammatory conditions, Tregs have been shown both to secrete cytokines and to lose Foxp3 expression^7,11^. Despite stable Foxp3 expression of nTregs *in vitro*, we thought that over time NIKtg Tregs may lose Foxp3 expression *in vivo* and subsequently contribute to the lung pathology in mice when NIK is driven by Foxp3^Cre^. To track loss of Foxp3 expression *in vivo*, we crossed Foxp3^Cre^ mice to ROSA26^fl-STOP-YFP^ lineage tracing mice (hereafter referred to as Foxp3^Cre^/R26^YFP^). In these mice, once a cell expresses Foxp3 it is marked by permanent YFP expression, so that YFP^+^Foxp3^−^ expression defines cells that once expressed Foxp3, but subsequently lost it^7^. We bred Foxp3^Cre^/R26^YFP^ to NIKtg mice and assessed proportions of YFP^+^ cells that had retained or lost Foxp3 expression. The proportion of YFP^+^Foxp3^−^ (ex-Foxp3^+^) T cells in NIKtg mice was much larger than in WT littermate controls, and this population expanded over time in the blood (Fig. 7a,b). Up to about 8 months of age, the proportion of WT ex-Foxp3^+^ T cells hovered around 10% of YFP^+^ cells, while the proportion of NIKtg ex-Foxp3^+^ T cells climbed from ~20% to ~70% during this same time frame (Fig. 7b). Interestingly, the proportion of ex-Foxp3^+^ T cells in WT blood increased with age after 8 months as well, albeit more slowly and to a lesser magnitude than in NIKtg. At euthanasia, high proportions of NIKtg ex-Foxp3^+^ T cells also were evident in the spleen and lymph nodes (Fig. 7c). The increased proportion of NIKtg ex-Foxp3^+^ T cells was not accompanied by a concomitant loss in Foxp3^+^ cell numbers. Instead, both Foxp3^+^ and ex-Foxp3^+^ T cell numbers were higher in NIKtg compared to WT mice (Fig. 7d), but the latter were increased by a larger margin. Thus, we think it likely that the increased proportion and numbers of ex-Foxp3^+^ T cells are due to a combination of increased propensity to lose Foxp3 and increased proliferation of ex-Foxp3^+^ T cells. However, we cannot rule out that increased cell death in a population of NIKtg Foxp3^+^ Tregs could contribute to the shift towards ex-Foxp3^+^ T cells.

**Figure 8:**
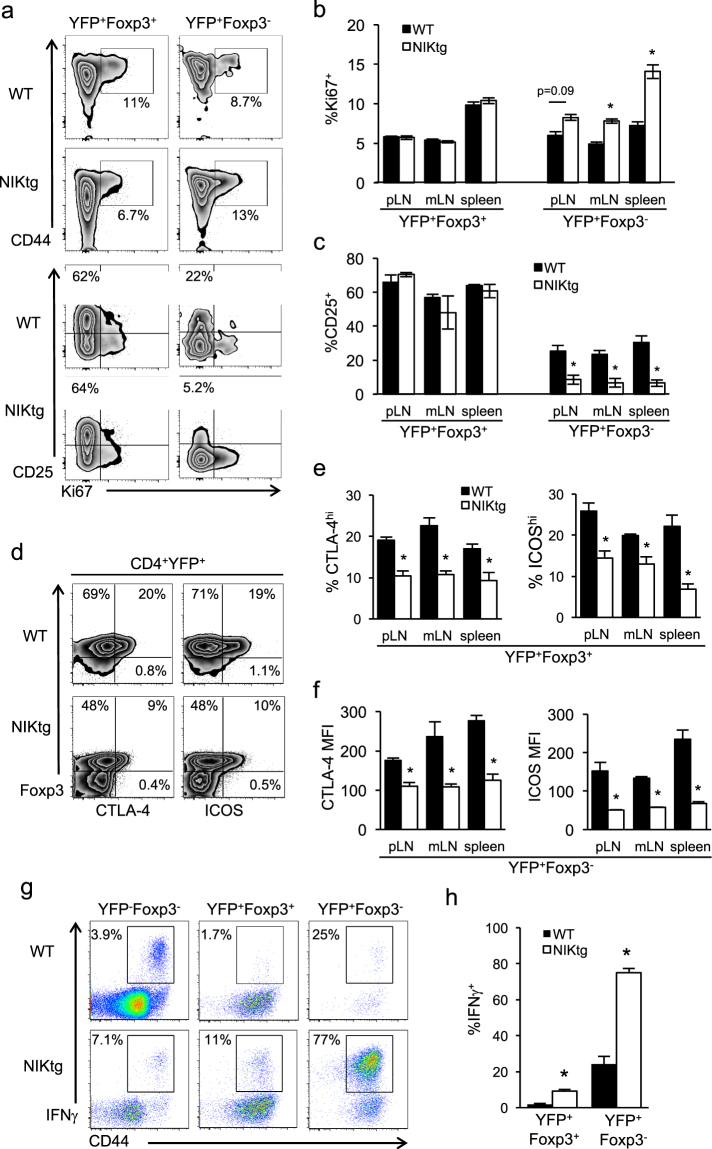
Constitutive NIK expression alters proliferation, phenotype, and cytokine production by ex-Foxp3^+^ T cells. Lymphocytes from NIKtg/Foxp3^Cre^/R26^YFP^ and WT/Foxp3^Cre^/R26^YFP^ littermates were gated on Tregs (CD4^+^YFP^+^Foxp3^+^) or ex-Foxp3^+^ T cells (CD4^+^YFP^+^Foxp3^−^) as in Fig. 7 and assessed for expression of the indicated markers directly *ex vivo* (**a–f**) or for expression of IFNγ after PMA + ionomycin stimulation (**g**,**h**). (**a**,**d**,**g**) Representative flow cytometry plots gated on live CD4^+^ lymphocytes plus additional markers as indicated. (**b**,**c**,**e**,**f**) Quantitation of indicated populations from pLN, mLN, and spleen. (**h**) Quantitation of IFNγ-producing T cells from spleen. Data are from one representative experiment of 3 (**c–h**) or one representative experiment of 2 (**a**,**b**); replicate data are shown in Supplemental Fig. S7. Bar graphs depict means +/− SD (n = 4 mice per group). *p < 0.05.

We surmised that NIK activation might provide a proliferative advantage to ex-Foxp3^+^ T cells. Indeed, Ki67 staining showed a higher proportion of ex-Foxp3^+^ T cells undergoing cell division in NIKtg compared to WT mice, but showed no proliferative difference between NIKtg and WT Foxp3^+^ cells (Fig. 8a,b). This difference reached statistical significance in one experiment and showed a trend in a second experiment (Fig. 8a top, b, and Supplementary Fig. S7). Surprisingly, the most proliferative Foxp3^+^ and ex-Foxp3^+^ T cells were those that had lost CD25 expression (Fig. 8a bottom). Similar proportions of WT and NIKtg Foxp3^+^ T cells expressed CD25, but nearly all NIKtg ex-Foxp3^+^ T cells lost CD25 expression while up to 30% of WT ex-Foxp3^+^ T cells retained it (Fig. 8a bottom, c). We also quantified changes in CTLA-4 and ICOS in NIKtg Foxp3^+^ and ex-Foxp3^+^ T cells. Similar to mixed BM chimeras, NIKtg Foxp3^+^ Tregs expressed less CTLA-4 and ICOS than did WT Foxp3^+^ Tregs (Fig. 8d,e). In ex-Foxp3^+^ T cells of both genotypes, CTLA-4 and ICOS decreased, but expression remained significantly lower in NIKtg vs. WT ex-Foxp3^+^ T cells (Fig. 8d,f).

Lineage tracing also allowed us to test the hypothesis that upon loss of Foxp3 expression, NIKtg T cells acquire pro-inflammatory effector function. Consistent with data from mixed BM chimeras, NIKtg Tregs in Foxp3^Cre^/NIKtg mice produced more IFNγ than their WT counterparts. In the ex-Foxp3^+^ population the effect was even more striking—over 75% of NIKtg ex-Foxp3^+^ T cells produced IFNγ (Fig. 8g,h). We also assessed IL-17, IL-4, and IL-9 production, but found no evidence for production of these cytokines (data not shown).

## Discussion

NIK is a key kinase downstream of costimulatory TNFR family members. NIK has been shown to be indispensible for acquisition of effector function and survival in conventional T cells^36,54–57^, but its effect in Tregs is unclear. Here, we report that constitutive NIK expression impairs Treg-mediated suppression of inflammation, resulting in autoimmune pathology. Treg impairment involves (i) decreased expression of Treg effector molecules and miRNAs necessary for Treg homeostasis and phenotypic stability, (ii) aberrant pro-inflammatory cytokine production by Tregs, and (iii) increased proportions and activation status of Tregs that have lost Foxp3 and acquired a pro-inflammatory phenotype.

Gene array experiments provided insight into the defective immunosuppressive properties conferred by constitutive NIK expression in Tregs. The global gene expression pattern in NIKtg Foxp3^+^ T cells clearly identifies them as Tregs. Fewer than 10% (77/832) of Treg signature genes showed expression levels that differed between NIKtg and WT Tregs. However, among those Treg signature genes that did differ between NIKtg and WT Tregs, a clear pattern emerged wherein genes known to be important to Treg fitness and regulatory function were disproportionately decreased in NIKtg Tregs. In most cases [e.g., *Ctla4*, *Nt5e* (CD73), *Ebi3* (IL-35 subunit), *Nrp1* (neuropilin), *Itgae* (CD103), *Tnfrsf9* (4-1BB), *Tnfrsf18* (GITR), and *Folr4* (folate receptor 4)], NIKtg Tregs retained expression of these genes above that of WT Tconv, but at lower levels than WT Tregs. In a few cases (e.g., *Cxcr3*, *Hif1a*, *Icos*, *Il10*, *Il10ra, Irf4*, and *Lag3*), Treg signature genes were unchanged or even decreased in NIKtg Tregs compared to WT Tconv. These changes are intrinsic to NIK expression in Tregs rather than secondary to an inflammatory environment since we performed the gene arrays on WT and NIKtg Tregs sorted from mixed bone marrow chimeras.

One Treg signature gene whose expression was unaffected by NIK in Tregs was Foxp3 itself. How do we reconcile normal Foxp3 expression levels with altered transcriptional profiles? Although Foxp3 is often described as the Treg master transcription factor, it is clear that Foxp3 does not directly repress or transactivate transcription of all Treg signature genes^58–60^. Thus, it is not surprising that we found many Treg effector genes downregulated in NIKtg Tregs despite normal levels of Foxp3. However, among genes shown to be direct targets of Foxp3-mediated transactivation^58^, several, including *Cd44*, *Ctla4*, *Icos*, and *Nrp1*, were downregulated in NIKtg vs. WT Tregs. Decreased expression of these genes, despite normal Foxp3 RNA and protein expression, could result from post-translational modifications of Foxp3, altered expression of transcription factors that interact with Foxp3, and/or chromatin modifications. Both acetylation and phosphorylation have been shown to alter Foxp3-mediated transactivation. For instance, TNFα signaling in inflamed joints activates protein phosphatase 1 (PP1), which de-phosphorylates Foxp3, leading to decreased Foxp3-mediated transactivation and suppressive function^61^. Notably, we found altered PP1 expression in NIKtg Tregs relative to WT Tregs by microarray. Foxp3 can both antagonize and cooperate with other transcription factors, including NF-AT and NF-κB. Foxp3 cooperates with NFAT downstream of TCR signaling to increase expression of *Ctla4* and *Cd25* in Tregs^52^, and *Nfatc1*, CTLA-4, and CD25 expression levels were all decreased in NIKtg Tregs. Conversely, Foxp3 normally prevents Treg production of pro-inflammatory cytokines, in part, via antagonizing the transcriptional activity of NF-AT and NF-κB at other loci^50^. Perhaps increased NF-κB activation in NIKtg Tregs overwhelms the Foxp3 transrepression capacity. Ratios of opposing transcription factors, rather than absolute expression levels of a particular transcription factor, determine fate decisions in other lymphocytes. For instance, the BLIMP/bcl6 ratio controls terminal effector vs. memory differentiation in B cells and T cells^62–64^. Thus, varying nuclear ratios of Foxp3 to other transcription factors could underlie altered function and phenotype in NIKtg Tregs despite normal Foxp3 expression. Supporting this idea, we found that NF-κB2, a positive feedback loop target of NF-κB, is increased in NIKtg Tregs. This suggests that despite Foxp3 expression, NF-κB activation in NIKtg Tregs is robust enough to induce gene transcription.

A non-mutually exclusive possibility is that chronic NIK expression alters Treg signature gene expression by mechanisms independent of NF-kB activation. For instance, NIK has been shown to increase CREB stability via phosphorylation-dependent and -independent mechanisms in hepatocytes^65^. In addition, IKKα—the primary target of NIK phosphorylation—can itself translocate to the nucleus and activate or repress gene expression via chromatin remodeling (e.g., phosphorylating histone H3) and via phosphorylation-dependent effects on co-activators and co-repressors^66^.

MicroRNA data from NIKtg Tregs suggested another mechanism by which chronic NIK activation could impair Treg-mediated suppression *in vivo*. Of 51 differentially regulated miRNAs, >75% were decreased in NIKtg Tregs vs. WT Tregs. A global decrease in miRNAs due to Dicer insufficiency intrinsically impairs Treg function and homeostasis and abrogates Treg suppressive function in inflammatory settings^47^. Moreover, specific miRNAs important in maintaining Treg homeostasis and suppressive function (10a, 125a, 146a, 155, 21) were all decreased in NIKtg Tregs^67–71^. Of these, miR-10a and miR-21 also help maintain Foxp3 expression in Tregs^68–70^, and miR-29 has been shown to restrain IFNγ production in Th1 cells^72–74^. Together, these data suggested that in addition to impairing their suppressive function, chronic NIK activation might also decrease Treg fitness and endow Tregs with pro-inflammatory capacity.

We tested this hypothesis and found that NIKtg Tregs do indeed make copious levels of the pro-inflammatory cytokines IL-2 and IFNγ and are under-represented in mixed BM chimeras. At first glance, this finding seems to contradict the observation of increased Treg numbers in Foxp3^Cre^/NIKtg mice (Fig. 7). However, the mixed BM chimeric mice remain healthy due to the presence of WT Tregs^36^, whereas Foxp3^Cre^/NIKtg mice experience significant inflammation-associated morbidity (Fig. 1). Tregs are well-known to expand in inflammatory conditions, and we previously showed that just prior to the early rapid demise of CD4^Cre^/NIKtg mice, Tregs are increased^36^. When WT Tregs are absent, the inflammatory environment causes some NIKtg Treg expansion, but the expanded Tregs are still insufficient to control the inflammation, and ultimately a proportion loses Foxp3. It is unclear at this time if chronic NIK activation actually increases the rate at which Tregs lose Foxp3 or expands ex-Foxp3^+^ T cells (as suggested by increased Ki67 staining). Based on the survival role of NIK and non-canonical NF-kB downstream of TNFR family members like BAFFR, it is also possible that chronic NIK activation rescues ex-Foxp3^+^ T cells from apoptosis, which would also explain the increased numbers of these cells relative to WT ex-Foxp3^+^ cells.

In summary, we found that constitutive NIK expression in Tregs causes transcriptional changes that serve to both impair Treg suppressive function and to give Tregs a pro-inflammatory phenotype. Under situations of normal host defense, activation of NIK downstream of costimulatory TNFRs on Tregs may be important to temporarily impede Tregs and allow elaboration of effective T cell immunity. However, if TNFR-mediated inflammatory signals become chronic, constitutive NIK expression in Tregs could impair negative feedback mechanisms and contribute to immunopathology, as we observed here. Moreover, when Foxp3 is lost from Tregs under conditions of constitutive NIK expression, those ex-Foxp3^+^ T cells are proliferative and capable of making pro-inflammatory cytokines. This is particularly dangerous given that Tregs are selected during development to be more autoreactive than Tconv.

Our findings are relevant to therapeutic strategies, already in clinical trials, that aim to treat autoimmunity and allograft responses by expanding Tregs *in vivo* or infusing *in vitro* expanded autologous Tregs^75–78^. These strategies depend on infused Tregs maintaining their suppressive identity, but under a chronic inflammatory state, this identity could be jeopardized by activation of NIK in Tregs. Thus, the level of NIK activation could be an important biomarker for Treg stability and predictor of treatment efficacy. Moreover, several TNF super family ligands and receptors have been linked to autoimmunity in GWAS studies^37,79,80^, and recently NIK was linked to MS in a GWAS-NR study^37^. Our results suggest that assessing the relationship between NIK activation and Treg suppressive function in patients with autoimmune diseases may provide evidence for NIK as a potential therapeutic target for these diseases.

## Materials and Methods

### Mice

Mice were housed under specific pathogen–free conditions at the Oregon Health and Science University animal facility. All procedures were approved by the OHSU Institutional Animal Care and Use Committee and were carried out in accordance with OHSU Animal Care and Use Program Standard Procedures. Thy1.1 (B6.PL-Thy1^a^/CyJ), CD45.1 (B6.SJL-Ptprc^a^Pepc^b^/BoyJ), CD4^Cre^ (B6.Cg-Tg(CD4-Cre)1Cwi/BfluJ), Foxp3^RFP^ (C57BL/6.Foxp3^tm1Flv/J^), Foxp3^Cre^ (NOD/ShiLt-Tg(Foxp3-EGFP/cre)1cJbl/J), and ROSA26^fl-STOP-YFP^ (B6.129 × 1-Gt(ROSA)26Sor^tm1(EYFP)Cos^/J) mice were from The Jackson Laboratory. NIKtg mice with a single copy NIK^fl-STOP-fl-GFP^ transgene knocked into the ROSA-26 locus were obtained from K. Rajewsky (Harvard Medical School, Boston, Massachusetts, USA)^81^. These mice are now available from the Jackson Laboratory (B6.Gt(ROSA)26Sor^tm5(Map3k14)Rsky^/J). All mice except Foxp3^Cre^ are on a C57BL/6 background. In all experiments using Foxp3^Cre^ mice, littermate control mice expressing Foxp3^Cre^, but not expressing the NIK transgene were used.

### Mixed bone marrow chimeras

Bone marrow (BM) was harvested from femurs and tibias of 11- to 18-day-old mice. Single-cell suspensions of BM were depleted of mature T cells via magnetic separation using anti-CD3-biotin. 2.5–10 × 10^5^ total BM cells were injected i.v. into lethally irradiated recipients. CD45.1 recipients were reconstituted with equal numbers of BM precursors from NIKtg/CD4^Cre^/Foxp3^RFP^ and WT/Thy1.1/Foxp3^RFP^ mice for use in *in vitro* Treg functional assays and microarrays. Thy1.1 recipients were reconstituted with equal numbers of BM precursors from NIKtg/CD4^Cre^ and WT/CD45.1 mice for use in phenotype and intracellular cytokine staining assays. T cells from mixed chimeras were used 8–16 weeks after reconstitution.

### Reagents and Antibodies

Recombinant IL-2, recombinant TGFβ, anti-IL-2 (54B6.1), and anti-IL-4 (11B11) blocking antibodies were from Peprotech. Retinoic acid was from Sigma-Aldrich. Anti-IFNγ blocking antibody (XMG1.2) was from BioXCell. Anti-CD3 (145–2C11), anti-CD28 (37.51) and Brefeldin A were from eBioscience. Fluorescently conjugated antibodies and other fluorescent reagents used for flow cytometry were anti-CD4 (RM4-5), anti-CD25 (PC61.5), anti-CD44 (IM7), anti-CD45.1 (A20), anti-CD103 (2E7), anti-CTLA4 (UC10-4B9), anti-Foxp3 (FJK-16S), rabbit anti-GFP (polyclonal), anti-rabbit (polyclonal), mouse anti-Ki67 (B56), anti-mouse IgG1 (M1-14D12), anti-IFNγ (XMG1.2), anti-IL-2 (JES6-5H2), anti-IL-4 (11B11), anti-IL-9 (RM9A4), anti-IL-17 (17B7), anti-ICOS (15F9), anti-Thy1.1 (cHIS51), CFSE, and Live/Dead Aqua. All staining antibodies were from eBioscience except anti-CD103 (BioLegend), anti-Ki67 and anti-IL-2 (BD Biosciences), and anti-GFP (Invitrogen). CFSE and Live/Dead Aqua were from Life Technologies. EasySep Negative Selection kits were from Stem Cell Technologies, and Foxp3 Fix/Perm Buffer sets and Mouse IL-2 ELISA kits were from BioLegend.

### Antibody staining and flow cytometry

Spleen cell suspensions and peripheral blood were red blood cell lysed and stained directly *ex vivo* or stimulated with PMA + ionomycin in the presence of Brefeldin A for 5 hours and then stained for surface markers and intracellular cytokines as described^82^. Cells were fixed and permeabilized per the manufacturer’s instructions (Biolegend Foxp3 Fix/Perm Buffer kit), and anti-cytokine antibodies were added at the same time as anti-nuclear antibodies (Foxp3 and Ki67). Cells were analyzed on an LSR II flow cytometer (BD Biosciences) and analyzed using FlowJo (Tree Star).

### TAT-Cre transduction and iTreg Differentiation

Magnetically purified CD4^+^CD25^−^ T cells (Tconv) from Foxp3^RFP^NIKtg and Foxp3^RFP^WT littermates were treated with recombinant TAT-Cre fusion protein to induce expression of the NIK transgene as previously described^36,83^. Briefly, CD4 T cells from NIKtg and WT littermate mice were incubated in serum-free media with 100 μg/ml TAT-Cre for 45 minutes, washed in serum-containing media, and cultured *in vitro* on anti-CD3– and anti-CD28–coated plates (5 μg/ml each) with 10 U/ml IL-2, 2–8 ng/ml TGF-β, 10 mM all-trans retinoic acid, 2 μg/ml anti–IFN-γ, and 1.4 μg/ml anti–IL-4 for 3-4 days. iTregs (CD4^+^Foxp3^RFP+^) were then sorted on a FACSAria to >98% purity. For NIKtg cultures, an additional GFP^+^ gate was used to sort those cells in which the NIK transgene had undergone Cre-mediated recombination.

### *In Vitro* Treg Functional Assay

NIKtg and WT Foxp3^RFP+^ Tregs were sorted from from iTreg cultures as described above or sorted directly from spleens of mixed BM chimeras (Fig. S2). Varying ratios of sorted Tregs were cultured with CFSE labeled CD45.1 (WT) CD4 Tconv, irradiated WT antigen-presenting cells, and anti-CD3 for 3 days as described^36^. Treg suppressive function was measured by analyzing CFSE dilution within the CD4 Tconv population.

### Cell sorting and RNA isolation for microarray and microRNA analyses

For each sample replicate, CD4 T cells were magnetically purified by negative selection from 3 pooled mixed BM chimera spleens. Cells were sorted on a FACSAria to isolate Tregs and Tconv from both NIKtg and WT populations. Sorting gates were as follows: NIKtg Tregs: CD4^+^Thy1.1^−^GFP^+^RFP^+^; NIKtg Tconv: CD4^+^Thy1.1^−^GFP^+^RFP^−^; WT Tregs: CD4^+^Thy1.1^+^GFP^−^RFP^+^; WT Tconv: CD4^+^Thy1.1^+^GFP^−^RFP^−^. RNA was extracted with RNeasy mini kit (Qiagen) and the samples were submitted to the Oregon Health and Science University Gene Profiling and Shared Resource for microarray and microRNA array analyses.

### Microarray analysis

Each RNA sample was labeled using the Ambion MessageAmp Premier RNA Amplification Kit and hybridized to an Illumina MouseRef 8 v2 Expression BeadChip Array. Image processing and expression analysis were performed using Illumina BeadArray Reader and GenomeStudio (v. 2010.1) Gene Expression module (v. 1.6.0) software. Intensity (.idat) files were produced by the BeadArray reader (Illumina scanner system). Intensity files, along with the BeadChip decode file (.dmap) were loaded to GenomeStudio’s Gene Expression module to generate probe/gene level signal intensity data (normalized and raw), perform QA/QC, and perform high level data visualization. Raw data were Lumi quantile normalized and log transformed before applying a 1.8 fold cutoff for differential regulation between WT and NIKtg Tregs. All microarray and microRNA array data are available at GEO with the reference number GSE80757 (http://www.ncbi.nlm.nih.gov/geo/query/acc.cgi?acc = GSE80757).

To generate lists of genes that are regulated between conventional CD4^+^ T cells and Tregs from the Immgen database, we used the population comparison tool and compared “T_4FP3 + 25 + _Sp” with “T_4FP3-_Sp”, submitted by T. Heng in the Benoist-Mathis lab, Joslin Diabetes Center^43^. We used Venny (http://bioinfogp.cnb.csic.es/tools/venny/index.html)^44^ to compare our list of genes that were regulated >1.8 fold between WT and NIKtg Tregs with the Immgen-generated lists of genes upregulated and downregulated in Tregs vs. Tconv. For GSEA analysis, we used these Immgen-generated lists of genes upregulated and downregulated in Tregs vs. Tconv as our gene sets. Enrichment of these gene sets in our microarray expression dataset was performed using the Gene Set Enrichment Analysis software from the Broad Institute^45,46^. The metric for ranking genes was log_2_ ratio of classes, and permutation type was gene set.

### MicroRNA array analysis

Each RNA sample was labeled using the Genisphere HSR miRNA Flash Tag Biotin protocol and hybridized to an Affymetrix GeneChip miRNA Array (v2). Image processing was performed using Affymetrix Command Console (AGCC) version 3.1.1 software, and analysis was performed using Affymetrix miRNA QC Tool version 1.1.1.0 software. Image processing of sample DAT files to generate probe intensity CEL files was completed in Affymetrix GeneChip Command Console (AGCC) version 3.1.1 software. Each array was analyzed using the Affymetrix miRNA QC Tool version 1.1.1.0 software to calculate the quality assessment metrics on non-normalized data. A multi-chip analysis incorporating all samples was performed according to the Genisphere FlashTag Biotin HSR labeling protocol manual (28Jan10). Probeset intensities were normalized using the Robust Multi-array Average (RMA) algorithm in Expression Console (build 1.4.1.46). Log_2_ intensities were averaged across treatment types, and log ratios (NIKtg Treg/WT Treg) were calculated and transformed to linear space before applying a 1.8 fold cutoff for differential regulation between WT and NIKtg Tregs.

### Statistics

Flow cytometric comparisons between NIKtg and WT T cell populations were assessed by Students t-test.

### Histology

Mouse organs were fixed with 10% (v/v) buffered formalin and embedded in paraffin. Sections were stained with hematoxylin and eosin and blindly scored by two pathologists.

### Data Availability

All microarray and microRNA array data are available at GEO with the reference number GSE80757 (http://www.ncbi.nlm.nih.gov/geo/query/acc.cgi?acc=GSE80757).

## Electronic supplementary material

Supplementary figures
