# Supplementary material for: Constitutive expression of NF-κB inducing kinase in regulatory T cells impairs suppressive function and promotes instability and pro-inflammatory cytokine production
Source: Sci Rep. 2017 Nov 7;7:14779. doi: 10.1038/s41598-017-14965-x (PMC5677020; doi:10.1038/s41598-017-14965-x)
Supplement: Supplementary file 1 — Supplementary figures [file 41598_2017_14965_MOESM1_ESM.pdf]

Title:
Constitutive expression of NF-kB inducing kinase in regulatory T cells impairs suppressive function and promotes instability and pro-inflammatory cytokine production

Authors:
Fanny Polesso[1†], Minhazur Sarker[1†], Arian Anderson[1], David C. Parker[1], and Susan E. Murray[1,2,*]

[1]Department of Molecular Microbiology and Immunology, Oregon Health & Science University, Portland, OR 97239
[2]Department of Biology, University of Portland, Portland, OR
[†]Contributed equally to this work

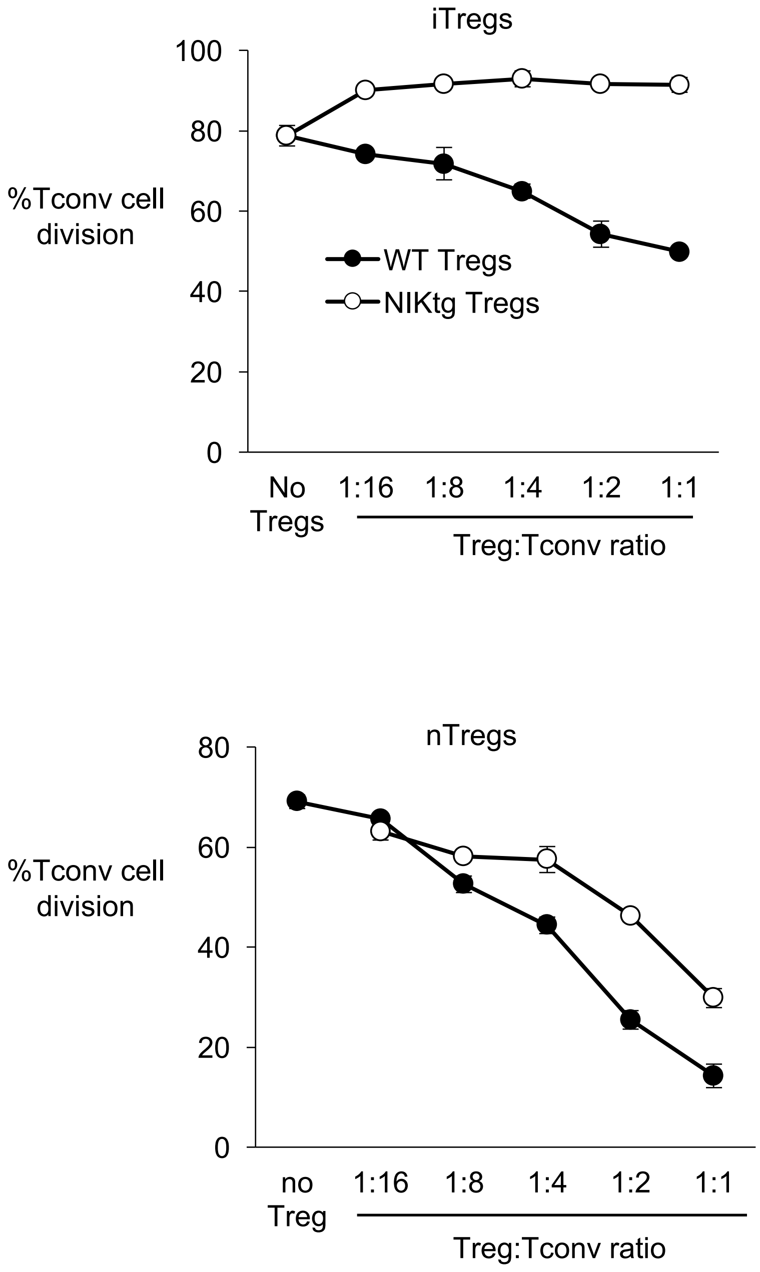

Supplementary Fig. S1. This figure shows repeat data for the experiments shown in Fig. 1, a-d. Top, inhibition of WT Tconv cell proliferation by WT vs. NIKtg iTregs. Bottom, inhibition of WT Tconv cell proliferation by WT vs. NIKtg nTregs.

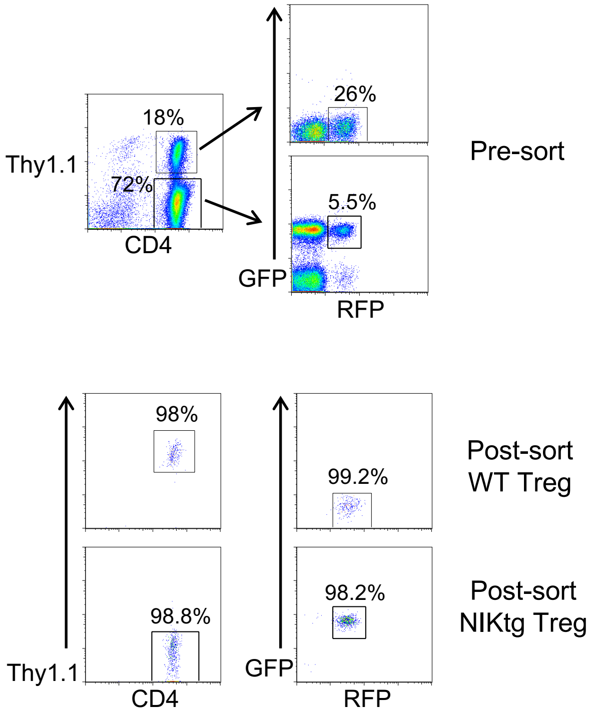

Supplementary Figure S2. Sort purity for NIKtg and WT Tregs from mixed bone marrow chimeras. CD4 T cells were magnetically enriched from NIKtg/CD4-Cre/Foxp3^{RFP} + WT/Thy1.1/Foxp3^{RFP} mixed chimeric spleens and then FACS-sorted on the basis of Thy1.1, CD4, RFP and GFP (NIK transgene expressed).

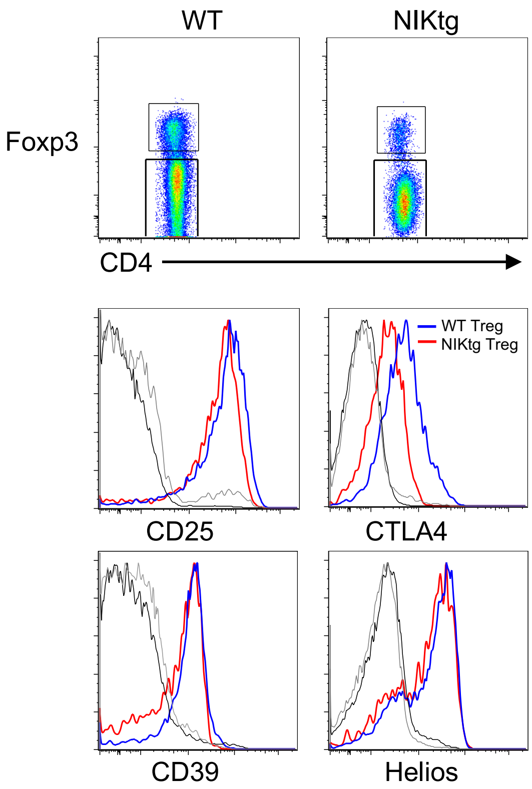

Supplementary Figure S3. NIKtg Tregs are Foxp3⁺ and express other Treg markers. Top, Foxp3 expression on CD4 gated NIKtg and WT splenocytes from mixed BM chimeras created by reconstituting lethally irradiated mice with a 50:50 mix of $CD4^{Cre}$xNIKtg BM and congenically marked WT BM. Middle and bottom, expression of the indicated Treg markers on WT (blue) and NIKtg (red) $Foxp3^{+}$ Tregs gated as shown in the top panels. Expression of these markers on WT (black) and NIKtg (gray) Tconv is shown for comparison.

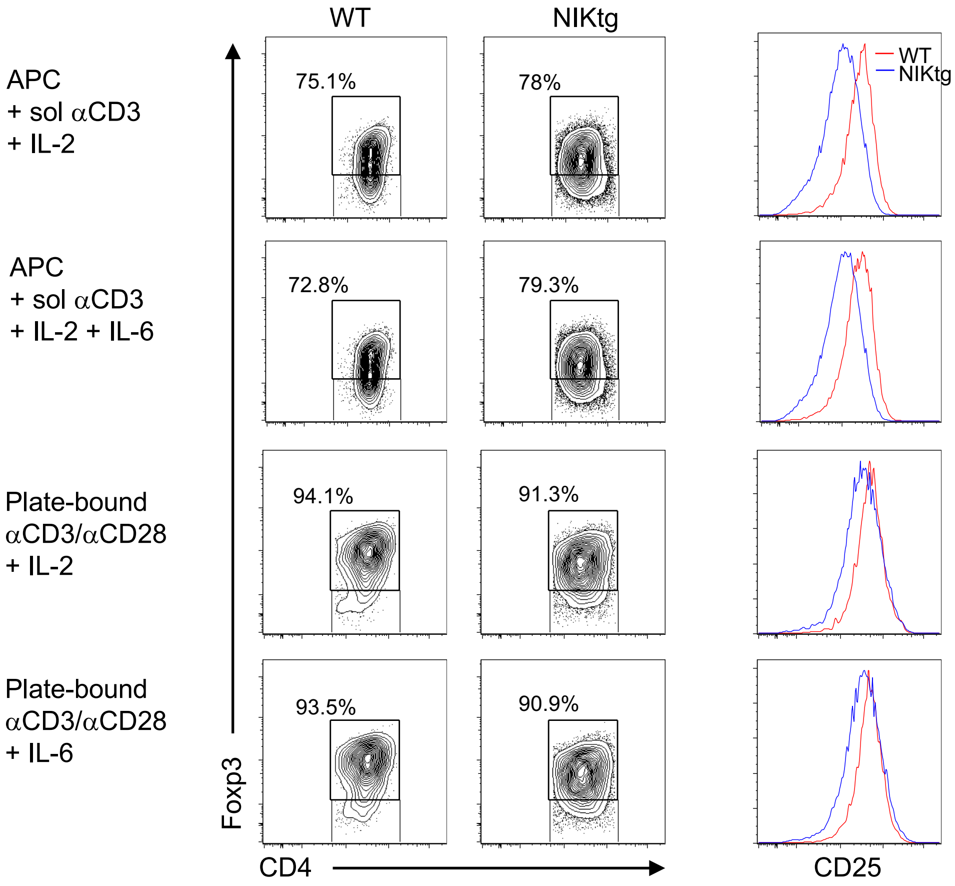

Supplementary Figure S4. NIKtg and WT nTregs display equally stable Foxp3 expression during short term in vitro culture, but NIKtg Tregs have decreased CD25 expression. WT and NIKtg Foxp3^{RFP+} Tregs were sorted from spleens of mixed BM chimeras created by reconstituting lethally irradiated mice with a 50:50 mix of CD4^{Cre}xNIKtg BM and congenically marked WT BM. They were then cultured under the conditions indicated above, and assessed for Foxp3 and CD25 expression 6 days later. APC, antigen presenting cells, which consisted of congenically labeled WT splenocytes irradiated with 10 Gy. Soluble αCD3 was used at 5ug/ml. Plate-bound αCD3 and αCD28 were coated at 5ug/ml and 2ug/ml, respectively. IL-2 and IL-6 were used at 100U/ml and 20ng/ml, respectively.

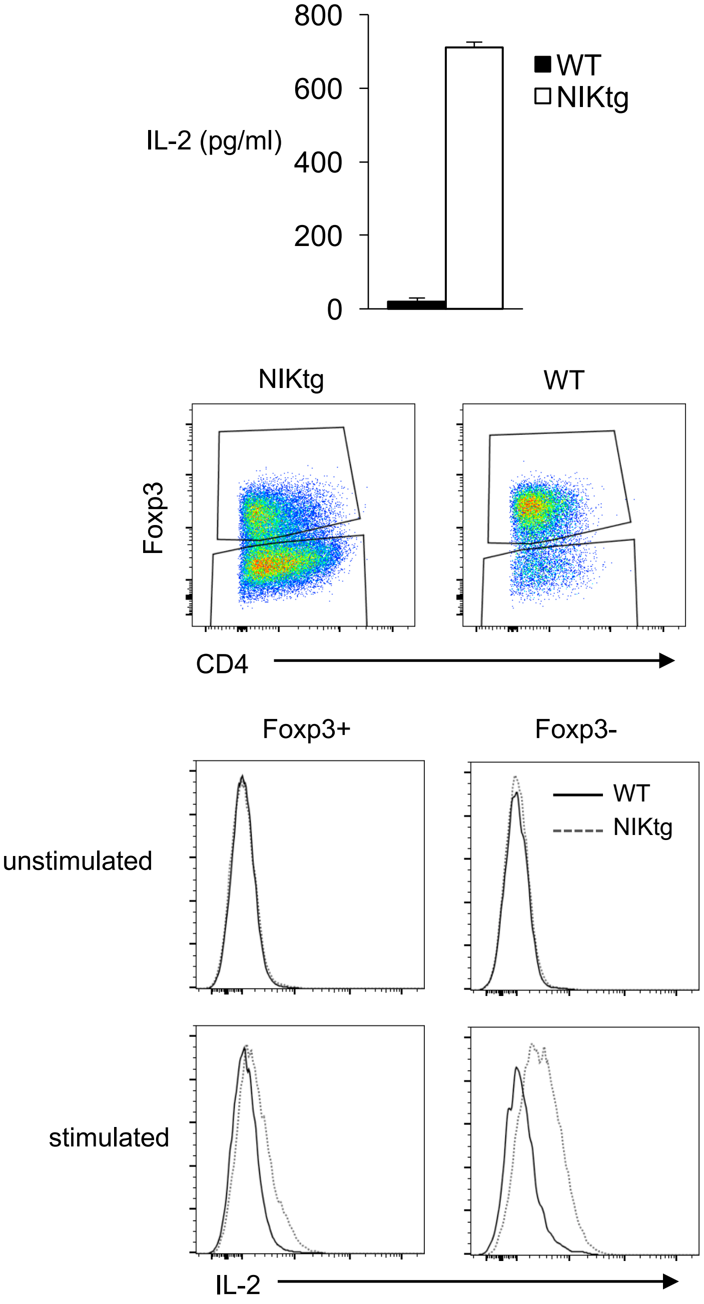

Supplementary Figure S5. This figure shows repeat data for the experiments shown in Fig. 5, e-f. NIKtg and WT T cells were differentiated under iTreg inducing conditions, sorted on the basis of Foxp3-RFP, then recultured for an additional 3 days in the absence of exogenous IL-2. Top, IL-2 concentration in secondary culture supernatants. Middle and bottom, Foxp3 expression and intracellular IL-2 production by $Foxp3^{+}$ and $Foxp3^{-}$ T cells upon PMA + ionomycin stimulation after secondary culture.

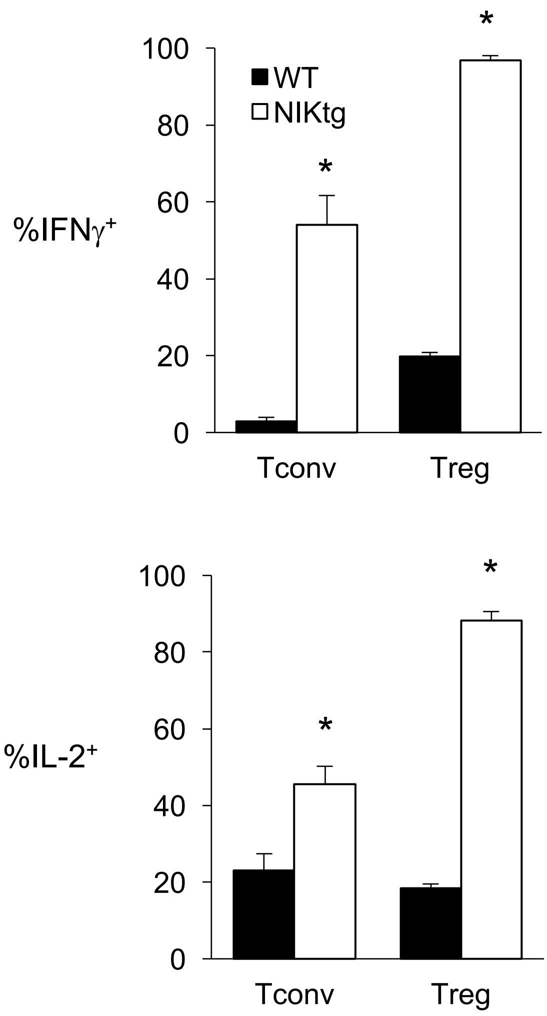

Supplementary Figure S6. This figure shows repeat data for the experiments shown in Fig. 6. NIKtg and WT $CD4^+Foxp3^+$ Treg and $CD4^+Foxp3^-$ Tconv from mixed BM chimeras were assessed for IFNγ and IL-2 production by intracellular cytokine staining upon PMA plus ionomycin stimulation.

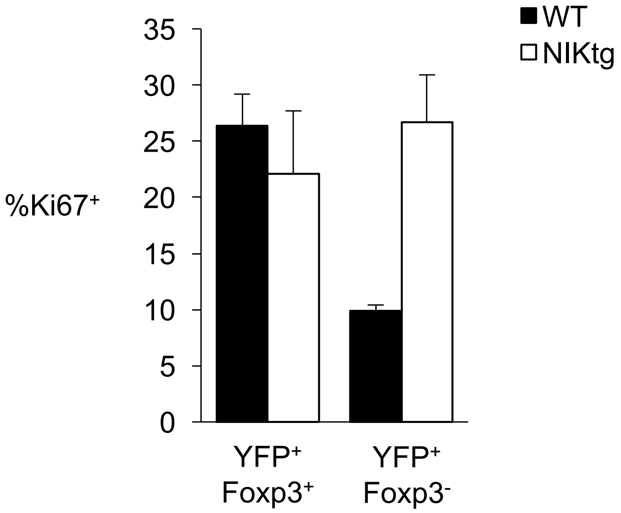

Supplementary Figure S7. This figure shows repeat data for the experiment shown in Fig. 8a-b. Splenocytes from NIKtg/Foxp3^{Cre}/R26^{YFP} and WT/Foxp3^{Cre}/R26^{YFP} littermates were gated on Tregs (CD4^{+}YFP^{+}Foxp3^{+}) or ex-Foxp3^{+} T cells (CD4^{+}YFP^{+}Foxp3^{-}) and assessed for Ki67 expression by intracellular flow cytometric staining.
